# Startup of Demo-Scale Anaerobic Digestion Plant Treating Food Waste Leachate: Process Instability and Recovery

**DOI:** 10.3390/ijerph19116903

**Published:** 2022-06-05

**Authors:** Seung Gu Shin, Su In Kim, Seokhwan Hwang

**Affiliations:** 1 Department of Energy Engineering, Future Convergence Technology Research Institute, Gyeongsang National University, 33 Dongjin-ro, Jinju 52828, Korea; sgshin@gnu.ac.kr; 2 Department of Energy System Engineering, Gyeongsang National University, 33 Dongjin-ro, Jinju 52828, Korea; 3 Division of Environmental Science and Engineering, Pohang University of Science and Technology, 77 Cheongam-ro, Pohang 37673, Korea; skim02@postech.ac.kr; 4 Institute for Convergence Research and Education in Advanced Technology (I-CREATE), Yonsei University, 85 Songdogwahak-ro, Yeonsu-gu, Incheon 21983, Korea

**Keywords:** food waste leachate, anaerobic digestion, organic loading rate, sequencing batch reactor, recovery, real-time PCR

## Abstract

A demo-scale (600 m^3^ working volume) anaerobic digester treating food waste leachate was monitored during its startup period. The operation strategy was adjusted twice (i.e., three distinct phases) during the operation to recover the process from instability. During the first phase, the organic loading rate (OLR) > 2.7 kg chemical oxygen demand (COD)/m^3^∙day corresponded to volatile fatty acid (VFA) accumulation along with a decreasing pH, resulting in the drop in biogas yield to 0.43 ± 0.9 m^3^/kg COD_in_. During phase 2, fast recovery of this process was aimed at using a sequencing batch operation. One batch cycle (5 to 2 days) consisted of the combined drawing and feeding step (5 h), the reacting step (91 to 17 h), and the settling step (24 h). The duration of the reacting step was determined for each cycle such that (1) the biogas production ceased before the cycle end and (2) the residual VFA concentration was < 1 g/L. In total, 11 cycles were operated with a gradual increase in biogas yield to 0.55 m^3^/kg COD_in_ with the absence of any sign of system disturbance. After phase 2, the digester was fed at the designed OLR of 4.1 ± 0.3 kg COD/m^3^∙day. The biogas yield was elevated to 0.58 ± 0.2 m^3^/kg COD_in_ during phase 3 with the residual VFA concentration maintained at 2.2 ± 0.6 g/L. Methanogen populations, as determined by real-time PCR, did not change significantly throughout the period. These results imply that the adaptation of this process to the OLR of ca. 4 kg COD/m^3^∙day was not due to the increase in methanogen population but due to the elevation of its activity. Overall, this study suggests that the sequencing batch operation with adjustable cycle duration can be one successful recovery strategy for biogas plants under system instability.

## 1. Introduction

About one-third of food materials are discarded as food waste globally. The recycling of food waste has gained more attention recently; however, a significant stream of secondary waste, namely food waste-recycling wastewater or food waste leachate (FWL), is generated during the recycling process [1]. FWL has high levels of organic materials derived from food waste [2]. Therefore, the treatment of FWL in an eco-friendly way is desirable.

Anaerobic digestion has been widely applied to process wastes and wastewaters from agro-industries [3]. Anaerobic digestion is a biological process that involves a series of biochemical reactions mediated by different microbial groups. These groups of microorganisms are broadly divided into hydrolyzing/acid-producing bacteria and acid-consuming consortia; the latter are mainly composed of syntrophic bacteria and methanogens. With an easily biodegradable feedstock, the former generally shows a higher growth and substrate utilization rate than the latter [4]. The intermediate acids can accumulate when the activities of the two groups diverge, e.g., in response to the increase in OLR and/or the inhibition of syntrophs/methanogens. The accumulation of acids may lead to process instability by decreasing the fitness of the methanogenic consortia and/or disturbing the favorable pH range (6.5–8.2) of methanogens [5].

When an anaerobic digester suffers from instability, the desired function of reducing pollutants and producing biogas cannot be met at the designed capacity. Such an episode may not only reduce the economic feasibility of a biogas facility but also damage the reputation of the biogas industry. Therefore, it is important to recover an unstable process as fast as possible with minimal uncertainty. However, there are only a limited number of actions that the operator can employ in an unstable process, such as decreasing the loading rate (stopping or decreasing feeding, diluting feedstock) [6], supplying external seed sludge (bioaugmentation) [7], modifying the process parameters [8], diversifying the feedstock (co-digestion) [9], and supplying trace elements [10]. Among these, stopping feeding is one of the most frequently practiced recovery methods because it is simple and does not require further preparation. In principle, stopping feeding means the process returns back to the stage of the startup period. A stepped increase in loading by increasing the flow rate is usually employed in this stage, but it takes a long time to reach the designed loading rate [11].

A continuous or semi-continuous feeding operation, such as a continuous stirred tank reactor (CSTR), is conventionally employed to process wastewaters with high solids content such as sewage sludge and FWL [12]. Previous studies have shown that anaerobic SBR, employing the four steps of feeding, reacting, settling, and drawing, is also feasible to treat such wastewaters [13]. The SBR operation has advantages over the continuous operation for maintaining a shorter hydraulic retention time (HRT) and higher organic loading rate (OLR) by retaining a high biomass concentration during the settling and drawing steps [14]. The biomass concentration is one of the critical factors that govern the startup period and recovery time. Therefore, SBR operation during the recovery of an unstable CSTR digester could be beneficial to reduce the recovery time.

This study was conducted to test a modified SBR operation for a digester under unstable conditions. The feature of the modified SBR operation was that the duration of each cycle during SBR was adjusted, unlike typical anaerobic SBR with a fixed cycle time [15], to facilitate recovery operation. We hypothesized that this operation would accelerate the process recovery by containing most of the anaerobic consortia within the system during the settling process without facing the risk of acidification by allowing enough or “on-demand” reaction time to complete the anaerobic pathway to methanogenesis. Here, system instability was defined as a state with high (>5 g/L) volatile fatty acid (VFA) concentrations and/or decreasing pH, while stability indicators were assumed as lower (<3 g/L) VFA and stable pH levels. The demo-scale (600 m^3^ working volume) anaerobic digester treated FWL as the sole feedstock. Process parameters along with methanogen populations were monitored during the recovery period.

## 2. Materials and Methods

### 2.1. Demo-Scale Anaerobic Digester

**Figure 1:**
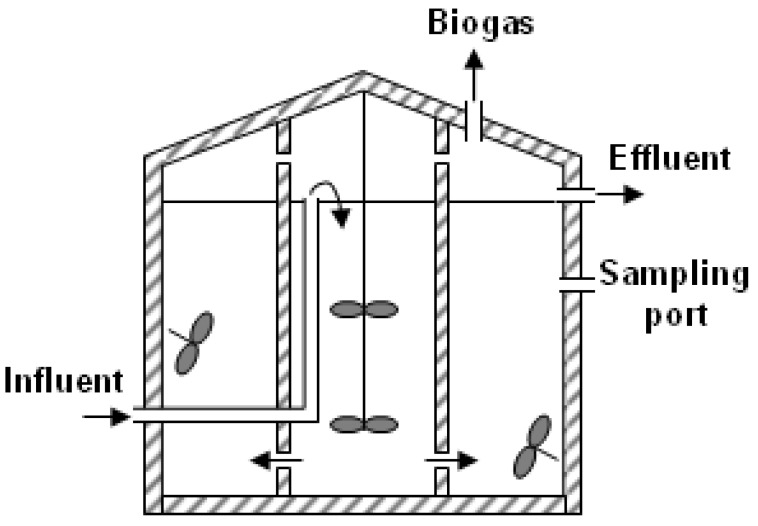
The schematic of the demo-scale anaerobic digester. Black arrows indicate the flow regime.

The design and operation of the demo-scale anaerobic digester were based on a previous lab-scale study [12]; the design HRT of 20 days was employed. A cylindrical 600-m^3^ anaerobic digester (diameter of 9.5 m and a wet-volume height of 8.5 m) was built on a local landfill site in Korea. The digester consisted of internal (150 m^3^ working volume) and external (450 m^3^) cylinders, where the former receives a new substrate (Figure 1). Mechanical mixing was conducted with a top-mount mixer in the internal cylinder and two side-entry mixers in the external cylinder. The digester was initially filled with anaerobic seed sludge (16.6 g volatile solids (VS)/L) from a full-scale anaerobic digester. The FWL was collected regularly from a local food waste-recycling facility and processed before injection by a 10 mm drum screen to reject non-digestible wastes. The FWL contained high organic content as represented by 96.0 ± 19.5 g chemical oxygen demand (COD)/L (*n* = 49; Table 1). The digester was agitated continuously with mechanical mixers except during the SBR operation. The temperature was kept at 35 ± 1 °C throughout the experimental period using internal circulation for heat exchange. No pH correction was made in the digester.

**Figure 2:**
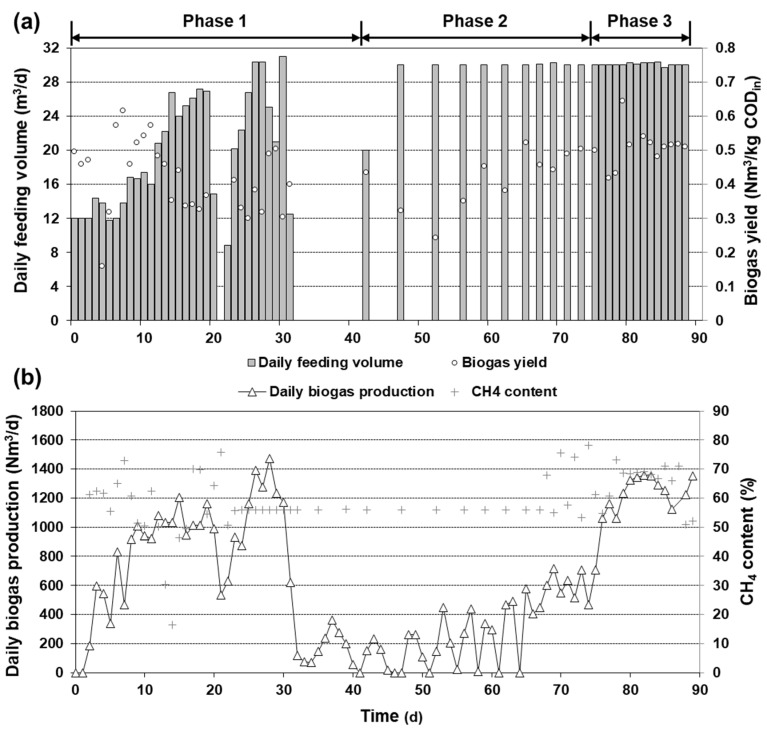
Profiles of (**a**) daily feeding volume, biogas yield, and (**b**) daily biogas production, CH_4_ content feeding volume during the operation.

The overall experimental period of 89 days was divided into three phases. In phase 1 (0–41 days), a gradual increase in the feeding volume from 12 m^3^/day (40% of the aimed rate) to 30 m^3^/day was planned. Feeding of 12 m^3^/day at 0 day corresponded to an OLR of 1.68 kg COD/m^3^∙day (based on the temporal COD value of FWL) and a feed-to-microorganism ratio of 0.101 kg COD/kg VS(∙day). Later, the digester became unstable during the ramp-up operation (see Section 3.1 for details) and the feeding was stopped for process recovery during 32–41 days (Figure 2a).

**Figure 3:**
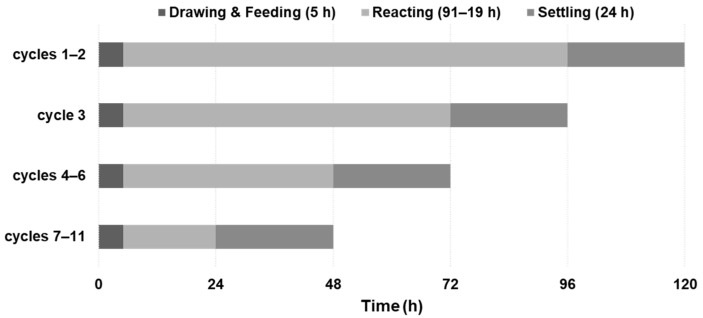
SBR operation with adjustable cycle duration during phase 2.

In phase 2 (42–74 days), an SBR operation was conducted as an alternative to the ramp-up feeding. Three steps constituted one cycle: drawing and feeding, reacting, and settling (Figure 3). The duration of the drawing and feeding step was 5 h; that of the settling step was 24 h. On the other hand, the duration of the reacting step was set as 91 h (cycles 1 and 2), 67 h (cycle 3), 43 h (cycles 4–6), and 19 h (cycles 7–11; see Section 3.2 for details).

In phase 3 (75–89 days), the operation mode was set back to semi-continuous feeding. The design capacity of 30 m^3^/day feeding was maintained in this period (Figure 2a).

### 2.2. Analytical Methods

Biogas production was monitored online using an automated gas metering system. The biogas CH_4_ content was determined using a portable analyzer (Biogas 5000, Geotech, Canada). Wet samples were collected daily from a port located at 6 m in height of the vessel. The pH, COD, total solids (TS), VS, total suspended solids (TSS), and volatile suspended solids (VSS) were determined according to the procedures in Standard Methods [16]. VFAs (C_2_–C_6_) and ethanol were measured using a gas chromatograph (6890 plus, Agilent, USA) equipped with an Innowax capillary column and a flame ionization detector. Total nitrogen and total phosphorus were measured using colorimetric test kits (TN-H and TP-H, C-Mac, Korea). Whole DNA was extracted and a real-time polymerase chain reaction (PCR) targeting the 16S rRNA gene of methanogen populations was performed as previously described [17].

## 3. Results

### 3.1. Startup and Process Instability (Phase 1)

**Figure 4:**
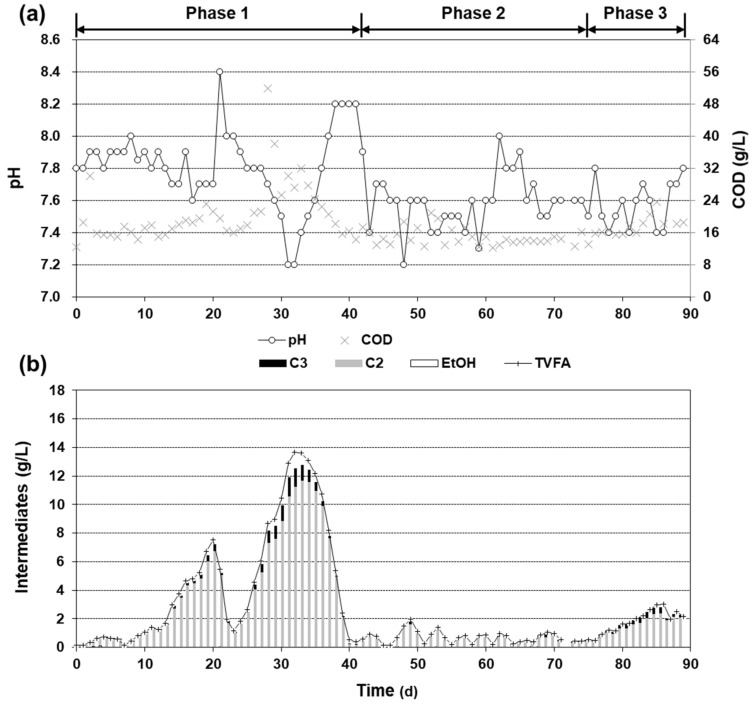
Profiles of (**a**) pH, COD, and (**b**) intermediates during the operation. TVFA here indicates the sum of VFAs (C_2_ to C_6_) and ethanol (EtOH).

The operational parameters of the demo-scale digester are summarized in Figure 2 and Figure 4. The digester was initially fed at a ratio of 12 m^3^/day (Figure 2a), which corresponds to an HRT of 50 days. The pH was maintained at 7.8 ± 0.1 (Figure 4a) with a residual TVFA (total VFA; including ethanol) concentration <0.5 g/L (Figure 4b) and a biogas yield of 0.47 ± 0.12 Nm^3^/kg COD_in_ (Figure 2a). The average CH_4_ content was 60.1% (Figure 2b).

Feeding was gradually elevated to 30 m^3^/day to meet the design HRT of 20 days during phase 1 (0–41 days). Although the previous study showed the stable operation of the lab-scale system at an HRT of 20 days [12], the demo-scale digester experienced system instability during this phase. At a daily feeding > 16 m^3^, corresponding to an OLR > ca. 2.7 kg COD/m^3^∙day, the residual TVFA concentration increased steadily in the demo-scale digester. The VFA accumulated up to 13.6 g/L (86% acetate and 8% propionate) on day 32 (Figure 4b), which was higher than the TVFA level in the substrate (Table 1). The increase in VFA concentration coincided with the drop in pH down to 7.2 (Figure 4a). Biogas yield also decreased to 0.38 ± 0.08 Nm^3^/kg COD_in_ (Figure 2a). This series of changes in the process parameters was commonly observed previously under unstable conditions [18,19].

Consequently, the feeding was stopped on day 32 to avoid acidification of the digester, which would further disturb anaerobiosis [6]. During the non-feeding period of 32–41 days, the TVFA concentration decreased to <0.5 g/L and the pH increased to 8.2 (Figure 4). Although these stability indicators (pH and TVFA) were recovered, the daily biogas production was below 400 Nm^3^/day during this period, reflecting the conversion of accumulated organics such as VFAs into biogas (Figure 2b).

### 3.2. Process Recovery with SBR Operation (Phases 2 and 3)

The SBR operation was employed to recover biogas yield during phase 2 (42–75 days). The schematic of the SBR operation is described in Figure 3. The duration of each cycle during SBR was adjusted, unlike typical anaerobic SBR with a fixed cycle time [15]. The duration of the drawing and feeding step was fixed at 5 h according to the speed limit of the feeding pump (6 m^3^/h). The duration of the settling step (24 h) was determined from a pre-test to maximize biomass settling (data not shown). The digester was not mixed during the settling and drawing and feeding steps. The duration of the reacting step, during which the digester was continuously mixed, was determined such that (1) the biogas production would cease before the cycle ends, i.e., before the next drawing and feeding step, and (2) the residual TVFA concentration would be <1 g/L. The former criterion was chosen to ensure enough bioconversion of the FWL fed at each cycle, while the latter criterion was considered as a stability indicator. Following these criteria, the duration of the reacting step decreased from 91 to 19 h during the eleven SBR cycles (Figure 2a and Figure 3). Thereby, the duration of each cycle was 5 days for cycles 1–2, 4 days for cycle 3, 3 days for cycles 4–6, and 2 days for cycles 7–11.

During phase 2, the TVFA concentration was kept low (<2 g/L at all points; <1 g/L at cycle ends), and the biogas yield increased gradually to 0.50 Nm^3^/kg COD_in_ (Figure 2a and Figure 4b). After day 75 (phase 3), the demo-scale anaerobic digester was operated at the designed HRT of 20 day with continuous feeding and mixing (phase 3; Figure 2a). The OLR in this phase was 4.1 ± 0.3 kg COD/m^3^∙day. In contrast to the results in phase 1, however, the biogas yield was elevated to 0.51 ± 0.02 Nm^3^/kg COD_in_ with the residual TVFA maintained at 2.2 ± 0.6 g/L.

### 3.3. Methanogen Populations

The 16S rRNA gene concentrations of methanogen orders (Methanobacteriales, Methanococcales, Methanomicrobiales, and Methanosarcinales) were determined using real-time PCR (Table 2). During phase 1, the total methanogen population was 2.0 ± 0.5 × 10^8^ copies/mL with Methanomicrobiales (69%) and Methanosarcinales (24%) as the major groups. The methanogen levels during phases 2 and 3 were quantified as 2.1 ± 0.6 × 10^8^ and 1.9 ± 0.3 × 10^8^ copies/mL, respectively, with similar compositions of the three groups as shown in phase 1. Overall, methanogen populations showed no significant changes during the whole observation period. Methanococcales were not detected in this study.

## 4. Discussion

In this study, the startup of a demo-scale anaerobic digester was monitored for 89 days. The initial strategy for the startup was a stepwise increase in the loading, which is a typical strategy to operate an anaerobic digester during startup [20]. However, the digester faced instability during phase 1, as represented by a decreasing pH and biogas yield (Figure 2) as well as an increasing TVFA concentration (Figure 4). One of the reasons for this instability is that the organics in FWL consist mainly of easily biodegradable organics [2]. The AD of easily biodegradable feedstock is susceptible to an imbalance between rapid hydrolysis/acidogenesis and slower methanogenesis when fluctuation occurs in terms of organic loading and/or operation conditions [21,22,23].

A typical SBR operation involves different steps at fixed durations [13,15]. In phase 2 of this study, however, the cycle time decreased gradually along with the decrease in the reacting time (Figure 3). The duration of the reacting time was empirically adjusted based on the biogas production profile and the residual VFA concentration; the biogas production rate increased each cycle with the depletion of the VFA intermediate. For example, the biogas production from one batch input of substrate (30 m^3^; 5% of the working volume) ceased at 3.3 days after the onset of feeding in cycle 3 and at 1.6 days after the onset of feeding in cycle 11; the biogas production rate approximately doubled between the two cycles. A similar attempt was reported for a sequencing batch operation of a thermophilic biomethane production using acidified palm oil mill effluent [24]. A recommended HRT of 3 days could be achieved by testing a wide range of HRTs (i.e., 10–1 days).

In this study, the increase in the biogas production rate coincided with the increase in the biogas yield during phase 2 (Figure 2), recovering higher than the level without system disturbance in phase 1 (0–13 days; Table 3). This boosting of biogas production activity can be due to (1) the increased concentration of anaerobic consortia, including methanogens, and/or (2) the increased activity of anaerobic consortia [25]. Given that the methanogen populations at the order level remained unchanged in this digester (Table 2), the increase in biogas activity in phase 2 is mainly attributable to the elevation of the methanogenic activity. Acclimation to a new feedstock or a new inhibition environment has been shown to increase the specific methanogenic activity in previous studies [26,27]. Although the methanogenic activity was not measured in this study, acclimation to FWL has likely affected this change.

The CH_4_ yield was estimated as 0.20, 0.23, and 0.34 Nm^3^/kg COD_in_ in phases 1, 2, and 3, respectively (Table 3). Considering the ~20% COD retained in the effluent, these values were converted to 0.25, 0.29, and 0.43 Nm^3^/kg COD_rem_, respectively. Again, the gradual increase in the CH_4_ yield shows that the activity of the biomass increased and the fraction of used substrate was directed more to catabolism than anabolism with time in this system [28]. The CH_4_ yield during phase 3 was higher than the reported maximum of 0.35 43 Nm^3^/kg COD_rem_. This might be from the imperfect homogenization of the digester (Figure 1), where some organics were left from the previous phases and converted into biogas in phase 3. Another possibility is that the CH_4_ content data was overestimated, as we used a portable analyzer (Biogas 5000) rather than more precise equipment such as a gas chromatograph.

The design and operation of an anaerobic process are typically based on process parameters, such as HRT and OLR, derived from preceding studies [14,29]. With a fixed, designed HRT or OLR, however, the anaerobic process could experience a period of instability as was described in phase 1 in this study (Figure 2). Although some operational parameters (e.g., pH and biogas production) can be monitored online or immediately after sampling, monitoring parameters based on wet analysis, such as VFA measurement, can cause a delay in determination. The SBR operation criteria suggested in this study require the monitoring of both biogas production and VFA concentration, possibly limiting its application. However, this limitation can be circumvented by using a parameter that can be estimated more rapidly, such as the intermediate alkalinity determined by simple titration [30]. By employing rapidly-determinable parameters, the SBR operation with an adjustable cycle duration (phase 2) minimizes system disturbance and facilitates the recovery from instability by ascertaining biogas production and VFA depletion before inputting another batch of the substrate. The current strategy is not limited to the scale of the digester; given the higher environmental and economic impact of system failure, a large-scale digester can be considered to use this strategy to minimize potential instability bound to a fixed-rate operation. The effluent of the digester can be further treated by conventional wastewater treatment processes for discharge or converted into fertilizer depending on the legislative requirements, economic feasibility, and/or farmland availability.

## 5. Conclusions

The operation of a demo-scale anaerobic digester treating FWL was monitored during process instability and recovery periods. During the initial continuous operation, OLR >2.7 kg COD/m^3^∙d corresponded to the accumulation of VFA and the drop in biogas yield. The SBR operation with an adjustable cycle duration was employed for 11 cycles, resulting in the recovery of the biogas production rate and yield. After the SBR operation, the digester was operated continuously at the designed OLR of 4.0 ± 0.3 kg COD/m^3^∙day; the biogas yield recovered to be stable at 0.52 ± 0.02 Nm^3^/kg COD_in_. Methanogen populations did not change significantly throughout the operation. These results imply that the SBR operation with an adjustable cycle duration could be one successful recovery strategy for biogas plants under system instability. The use of rapidly-determinable parameters to track the status of the digester would be necessary for timely decision making.

## Figures and Tables

**Table 1:** The characteristics of the substrate monitored throughout the experiment.

Parameter	Unit	*n* *	Average	Standard Deviation
pH	-	46	3.5	0.3
TS	g/L	48	58.5	13.2
VS	g/L	48	49.7	11.6
TSS	g/L	7	34.1	12.6
VSS	g/L	7	31.2	12.2
COD	g/L	49	96.0	19.5
SCOD	g/L	49	58.3	12.1
Acetate	g/L	49	7.1	1.5
Propionate	g/L	49	0.2	0.1
Ethanol	g/L	49	5.5	2.6
Total nitrogen	g/L	6	2.1	0.3
Total phosphorus	g/L	6	0.3	0.1

* The analyses were conducted approximately every two days (for those with *n* = 46–49) or every two weeks (for those with *n* = 6–7) throughout the experiment.

**Table 2:** The methanogen population structures at different phases.

16S rRNA Gene (×10^7^ Copies/mL)	Phase 1 (*n* = 20)	Phase 2 (*n* = 33)	Phase 3 (*n* = 7)
Methanobacteriales	1.5 ± 0.4	2.1 ± 0.6	2.3 ± 0.3
Methanomicrobiales	13.8 ± 4.8	13.5 ± 5.0	11.6 ± 2.9
Methanosarcinales	4.8 ± 1.4	4.9 ± 1.6	5.2 ± 0.7
Sum	20.1 ± 5.0	20.5 ± 6.4	19.1 ± 2.5

**Table 3:** Summary of COD balance and biogas production during the experiment.

Parameter	Phase 1	Phase 2	Phase 3	Overall
COD input (kg)	69,340	23,448	32,080	124,868
COD output (kg)	12,706	4673	6301	23,680
COD removal (kg)	56,634	18,775	25,778	101,187
Biogas production (Nm^3^)	26,295	9816	15,059	51,170
Biogas yield (Nm^3^/kg COD_in_)	0.38 (0.47) *	0.42	0.51	0.42
CH_4_ production (Nm^3^)	13,764	5465	9993	20,222
CH_4_ yield (Nm^3^/kg COD_in_)	0.20 (0.30) *	0.23	0.34	0.24

* Derived from 0–13 days (before instability).

## References

[1] Kim H., Kim J., Shin S.G., Hwang S., Lee C. (2016). Continuous fermentation of food waste leachate for the production of volatile fatty acids and potential as a denitrification carbon source. Bioresour. Technol..

[2] Shin S.G., Han G., Lee J., Cho K., Jeon E.-J., Lee C., Hwang S. (2015). Characterization of food waste-recycling wastewater as biogas feedstock. Bioresour. Technol..

[3] Li Y., Chen Y., Wu J. (2019). Enhancement of methane production in anaerobic digestion process: A review. Appl. Energy.

[4] Meegoda J.N., Li B., Patel K., Wang L.B. (2018). A review of the processes, parameters, and optimization of anaerobic digestion. Int. J. Environ. Res. Public Health.

[5] Rhee C., Park S.-G., Kim D.W., Yu S.I., Shin J., Hwang S., Shin S.G. (2021). Tracking microbial community shifts during recovery process in overloaded anaerobic digesters under biological and non-biological supplementation strategies. Bioresour. Technol..

[6] Speece R.E. (1996). Anaerobic Biotechnology for Industrial Wastewaters.

[7] Tale V.P., Maki J.S., Struble C.A., Zitomer D.H. (2011). Methanogen community structure-activity relationship and bioaugmentation of overloaded anaerobic digesters. Water Res..

[8] Vazifehkhoran A.H., Shin S.G., Triolo J.M. (2018). Use of tannery wastewater as an alternative substrate and a pre-treatment medium for biogas production. Bioresour. Technol..

[9] Prapinagsorn W., Sittijunda S., Reungsang A. (2018). Co-digestion of napier grass and its silage with cow dung for bio-hydrogen and methane production by two-stage anaerobic digestion process. Energies.

[10] Zhu X., Yellezuome D., Liu R., Wang Z., Liu X. (2022). Effects of co-digestion of food waste, corn straw and chicken manure in two-stage anaerobic digestion on trace element bioavailability and microbial community composition. Bioresour. Technol..

[11] Gong H., Liu M., Li K., Li C., Xu G., Wang K. (2020). Optimizing dry anaerobic digestion at pilot scale for start-up strategy and long-term operation: Organic loading rate, temperature and co-digestion. Bioresour. Technol..

[12] Shin S.G., Han G., Lim J., Lee C., Hwang S. (2010). A comprehensive microbial insight into two-stage anaerobic digestion of food waste-recycling wastewater. Water Res..

[13] Atasoy M., Eyice O., Cetecioglu Z. (2020). A comprehensive study of volatile fatty acids production from batch reactor to anaerobic sequencing batch reactor by using cheese processing wastewater. Bioresour. Technol..

[14] Angenent L.T., Sung S.W., Raskin L. (2002). Methanogenic population dynamics during startup of a full-scale anaerobic sequencing batch reactor treating swine waste. Water Res..

[15] Jagaba A.H., Kutty S.R.M., Lawal I.M., Abubakar S., Hassan I., Zubairu I., Umaru I., Abdurrasheed A.S., Adam A.A., Ghaleb A.A.S. (2021). Sequencing batch reactor technology for landfill leachate treatment: A state-of-the-art review. J. Environ. Manag..

[16] Eaton A.D., Clesceri L.S., Greenberg A.E. (2005). Standard Methods for the Examination of Water and Wastewater.

[17] Shin S.G., Lee S., Lee C., Hwang K., Hwang S. (2010). Qualitative and quantitative assessment of microbial community in batch anaerobic digestion of secondary sludge. Bioresour. Technol..

[18] Stuckey D.C. (2011). Anaerobic baffled reactor (ABR) for wastewater treatment. Environmental Anaerobic Technology: Applications and New Developments.

[19] Kim M., Ahn Y.-H., Speece R.E. (2002). Comparative process stability and efficiency of anaerobic digestion; mesophilic vs. thermophilic. Water Res..

[20] Musa M.A., Idrus S., Hasfalina C.M., Daud N.N.N. (2018). Effect of organic loading rate on anaerobic digestion performance of mesophilic (UASB) reactor using cattle slaughterhouse wastewater as substrate. Int. J. Environ. Res. Public Health.

[21] Li D., Ran Y., Chen L., Cao Q., Li Z., Liu X. (2018). Instability diagnosis and syntrophic acetate oxidation during thermophilic digestion of vegetable waste. Water Res..

[22] Jo Y., Rhee C., Choi H., Shin J., Shin S.G., Lee C. (2021). Long-term effectiveness of bioaugmentation with rumen culture in continuous anaerobic digestion of food and vegetable wastes under feed composition fluctuations. Bioresour. Technol..

[23] Shin S.G., Han G., Lee J., Shin J., Hwang S. (2019). A snapshot of microbial community structures in 20 different field-scale anaerobic bioreactors treating food waste. J. Environ. Manag..

[24] Abd Nasir M.A., Jahim J.M., Abdul P.M., Silvamany H., Maaroff R.M., Mohammed Yunus M.F. (2019). The use of acidified palm oil mill effluent for thermophilic biomethane production by changing the hydraulic retention time in anaerobic sequencing batch reactor. Int. J. Hydrog. Energ..

[25] Lee C., Kim J., Shin S.G., O’Flaherty V., Hwang S. (2010). Quantitative and qualitative transitions of methanogen community structure during the batch anaerobic digestion of cheese-processing wastewater. Appl. Microbiol. Biotechnol..

[26] Xing B.-S., Han Y., Wang X.C., Cao S., Wen J., Zhang K. (2020). Acclimatization of anaerobic sludge with cow manure and realization of high-rate food waste digestion for biogas production. Bioresour. Technol..

[27] Park J.-H., Yoon J.-J., Kumar G., Jin Y.-S., Kim S.-H. (2018). Effects of acclimation and pH on ammonia inhibition for mesophilic methanogenic microflora. Waste Manag..

[28] Michaud S., Bernet N., Buffière P., Roustan M., Moletta R. (2002). Methane yield as a monitoring parameter for the start-up of anaerobic fixed film reactors. Water Res..

[29] Demirel B., Yenigun O., Onay T.T. (2005). Anaerobic treatment of dairy wastewaters: A review. Process Biochem..

[30] Sun H., Ni P., Angelidaki I., Dong R., Wu S. (2019). Exploring stability indicators for efficient monitoring of anaerobic digestion of pig manure under perturbations. Waste Manage.

